# Functional Validation of Rare Human Genetic Variants Involved in Homologous Recombination Using *Saccharomyces cerevisiae*

**DOI:** 10.1371/journal.pone.0124152

**Published:** 2015-05-04

**Authors:** Min-Soo Lee, Mi Yu, Kyoung-Yeon Kim, Geun-Hee Park, KyuBum Kwack, Keun P. Kim

**Affiliations:** 1 Department of Life Science, Chung-Ang University, Seoul, Korea; 2 Department of Biomedical Science, CHA University, Seongnam, Korea; CNR, ITALY

## Abstract

Systems for the repair of DNA double-strand breaks (DSBs) are necessary to maintain genome integrity and normal functionality of cells in all organisms. Homologous recombination (HR) plays an important role in repairing accidental and programmed DSBs in mitotic and meiotic cells, respectively. Failure to repair these DSBs causes genome instability and can induce tumorigenesis. Rad51 and Rad52 are two key proteins in homologous pairing and strand exchange during DSB-induced HR; both are highly conserved in eukaryotes. In this study, we analyzed pathogenic single nucleotide polymorphisms (SNPs) in human *RAD51* and *RAD52* using the Polymorphism Phenotyping (PolyPhen) and Sorting Intolerant from Tolerant (SIFT) algorithms and observed the effect of mutations in highly conserved domains of *RAD51* and *RAD52* on DNA damage repair in a *Saccharomyces cerevisiae*-based system. We identified a number of *rad51* and *rad52* alleles that exhibited severe DNA repair defects. The functionally inactive SNPs were located near ATPase active site of Rad51 and the DNA binding domain of Rad52. The *rad51-F317I*, *rad52-R52W*, and *rad52-G107C* mutations conferred hypersensitivity to methyl methane sulfonate (MMS)-induced DNA damage and were defective in HR-mediated DSB repair. Our study provides a new approach for detecting functional and loss-of-function genetic polymorphisms and for identifying causal variants in human DNA repair genes that contribute to the initiation or progression of cancer.

## Introduction

Genomic instability due to defects with DNA repair proteins causes various inherited genetic disorders in humans and also leads a generalized cancer predisposition. DNA damage, including double-strand breaks (DSBs) and cross-linkages, occurs spontaneously during the normal cell cycle and upon exposure to ionizing radiation or mutagenic chemicals [1, 2]. Defects in the DNA repair process lead to DNA damage, which in turn can cause cell cycle arrest, apoptosis, and tumorigenesis [1, 2]. Homologous recombination (HR) is a crucial metabolic pathway found in all organisms, and is involved in the maintenance of somatic genome integrity in the presence of DNA DSBs, DNA inter-strand crosslinks, or stalled DNA replication forks [3]. In meiosis, a highly regulated HR process mediates the exchange of genetic information between the maternal and paternal chromosomes to produce non-identical haploid germ cells [4–6].

Rad51 and Rad52 are key proteins involved in DSB repair by HR and are conserved between humans and yeast [7–9]. Rad51 is a eukaryotic homolog of the bacterial RecA protein, which mediates homology searching and DNA strand exchange activities that result in joint molecule (JM) formation mediated by presynaptic filaments between homologous chromatids [9, 10]. Rad52 plays a role in DSB repair (DSBR) as well as in single-strand annealing, a Rad51-independent DSBR pathway [11]. During DSBR, Rad52 stimulates HR, which facilitates the formation of Rad51-ssDNA nucleofilaments in the presence of replication protein A complex, composed of the subunits Rfa1, Rfa2, and Rfa3 [12, 13]. Because Rad52 functions to anneal homologous single strand DNA in second-end capture, i.e., synthesis-dependent strand annealing and single-strand annealing [14, 15], cells deficient for Rad52 exhibit a defect in HR. In turn, the importance of Rad51 in the HR pathway is highlighted by the tumor suppressor protein BRCA2, which is involved in breast and ovarian cancers, as well as other types of cancers [16, 17]. The localization of human Rad51 to the DNA DSB requires the formation of a BRCA1-PALB2-BRCA2 complex [18], wherein BRCA2 interacts with Rad51 to initiate the strand-invasion step [19–23]. The BRC repeat domain of BRCA2 stabilizes the Rad51-ssDNA complex by inhibiting DNA-dependent Rad51 ATPase activity [24, 25]. Loss of this control owing to BRCA2 or Rad51 mutations may lead to gross chromosomal rearrangements and increased susceptibility to cancers [26, 27].

Many human diseases have a strong genetic component, and human genetic studies have successfully determined the causes of numerous rare Mendelian disorders [28]. Missense mutations can result in fatal or serious Mendelian disorders, or can be slightly deleterious, effectively neutral, or beneficial [29]. However, the molecular basis underlying the function of causal variants that result in the manifestation of these diseases are still largely unexplained.

Although many mutations in DNA repair genes have been reported, it is difficult to determine their clinical significance. Here, we describe functional analysis of causal variants in human *Rad51* and *Rad52* to determine if these missense mutations also result in loss-of-function or lethality in *Saccharomyces cerevisiae*. Specifically, we analyzed the JMs and recombination products of *rad51* and *rad52* mutants with causal variants that are associated with deficient strand exchange activity of the HR pathway. We validated the findings for deleterious mutations associated with human *RAD51* and *RAD52* and assessed their possible complementary roles as DNA damage repair proteins using a model system.

## Results and Discussion

### Identification of Deleterious *rad51* and *rad52* Alleles in Humans and Yeast

**Fig 1:**
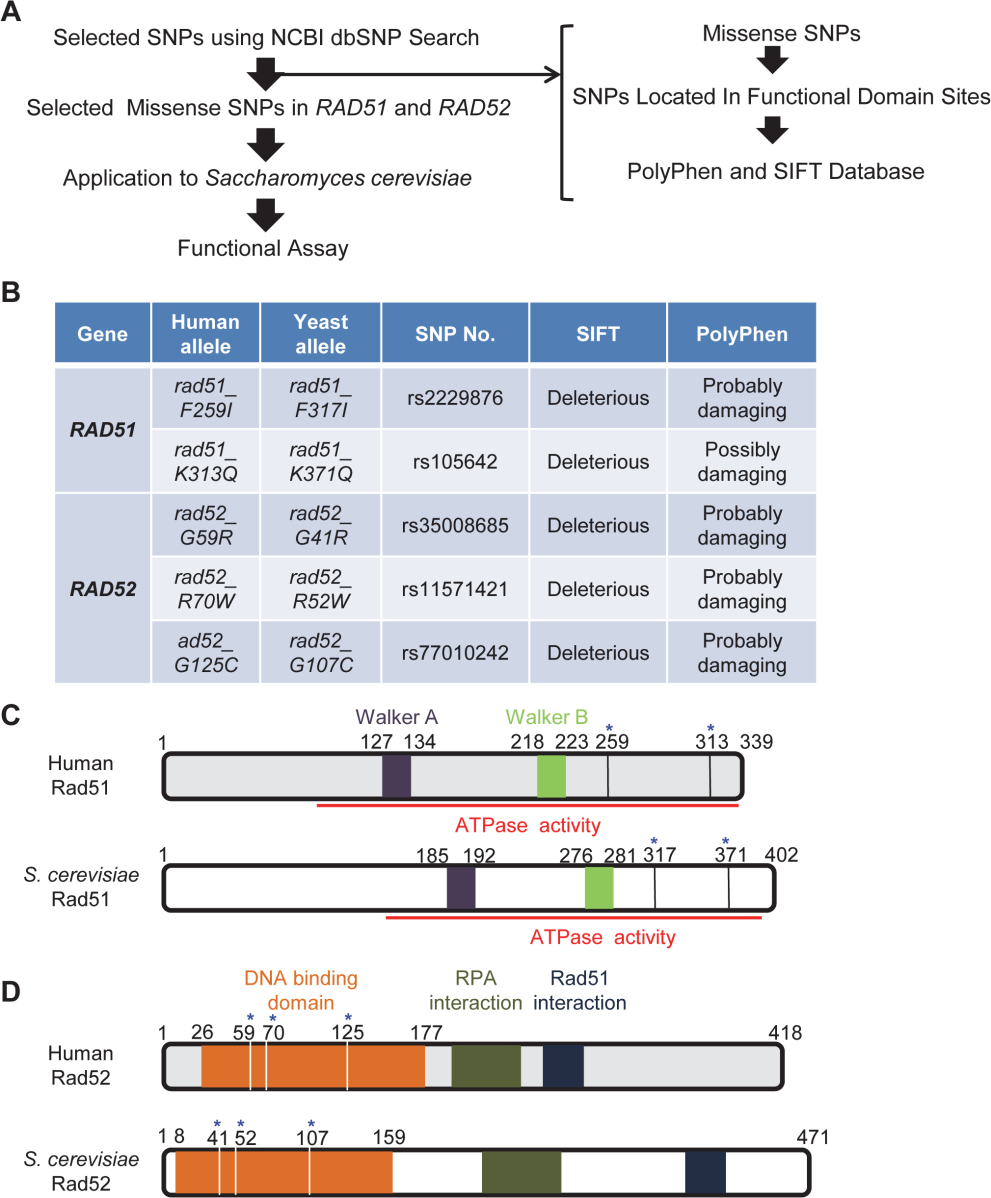
Schematic diagram of experiments and the selection of SNPs in human *RAD51* and *RAD52* genes. (A) Diagram indicating the method of SNP selection. The SNPs in *hRAD51* and *hRAD52* were selected using the National Center for Biotechnology Information Single Nucleotide Polymorphism Database (NCBI dbSNP), (http://www.ncbi.nlm.nih.gov/snp/). (B) Missense SNPs located in functional domains were compared with genetic variations using SIFT and PolyPhen. “Deleterious” and “Probably damaging” allelic mutants might affect function via structural alterations of Rad51 and Rad52. (C) Comparison of the functional domains of Rad51 in humans and yeast [30]. * regions containing *rad51-F317I* and *rad51-K371Q* mutations. (D) Comparison of the functional domains of Rad52 in humans and yeast [31, 32]. * regions containing *rad52-G41R*, *rad52-R52W*, and *rad52-G107C* mutations.

We selected 12 single nucleotide polymorphisms (SNPs) within *RAD51* (8 SNPs) and *RAD52* (4 SNPs) from the NCBI SNP database (S2 Table). We compared the homologs of these two genes in humans and yeast, but excluded several SNPs that were synonymous. The PolyPhen (http://genetics.bwh.harvard.edu/pph) and SIFT (http://sift.jcvi.org) programs were used to predict the effects of amino acid substitutions on protein function and structure, respectively (Fig 1A). From these analyses, we predicted that the *RAD51* SNPs, *rad51-F259I* and *rad51-K313Q*, and the *RAD52* SNPs, *rad52-G59R*, *rad52-R70W*, and *rad52-G125C* lead to the formation of non-functional proteins that would likely be non-functional in physiological conditions (Fig 1B). Specifically, these missense SNPs were located in the ATPase domains of Rad51 and DNA-binding domains of Rad52 that affect HR activity; these functional domains are highly conserved between humans and yeast (Fig 1C and 1D).

### Similarity of Functional Domains in Human and Yeast Genes

Rad51 and Rad52 protein sequences are highly conserved between human and *S*. *cerevisiae*. We aligned the Rad51 and Rad52 sequences in various species, including *Homo sapiens and S*. *cerevisiae*, *Pan troglodytes*, *Canislupus*, *Bos taurus*, *Rattus norvegicus*, *Mus musculus*, *Gallus gallus*, *Danio rerio*, *Kluyveromyces lactis*, and *Eremothecium gossypii* using the ClustalW program for multiple sequence alignment of proteins (S2 Fig). We evaluated deleterious polymorphisms in human *RAD51* and *RAD52* and compared equivalent mutations at the analogous positions in the corresponding yeast genes (Fig 1A and 1B). Human Rad51 and yeast Rad51 exhibit functional similarity and the Rad51 ATPase domains containing Walker A and Walker B motifs were identical (Fig 1C) [30]. For example, human *rad51-F259* and *K313* mutations, located in the ATPase activity region, corresponded to *S*. *cerevisiae rad51-F317* and *K371*, respectively (Fig 1C). Similarly, three *RAD52* SNPs (*rad52-G59R*, *rad52-R70W*, and *rad52-G125C*), which were selected using the NCBI SNP database, were located at the DNA binding site in both human and *S*. *cerevisiae* Rad52 proteins (Fig 1D). It has been reported that the DNA-binding, RPA, and Rad51 interaction regions are conserved in both the human and *S*. *cerevisiae* Rad52 domains (Fig 1D) [31, 32]. Thus, based on the human and *S*. *cerevisiae* Rad51 and Rad52 domains, we believe that the *rad51-F317I* and *rad51-K371Q* mutations affect the ATPase activity of Rad51 and that the *rad52-G41R*, *rad52-R52W*, and *rad52-G107C* mutations affect the DNA-binding activity of Rad52, respectively.

**Fig 2:**
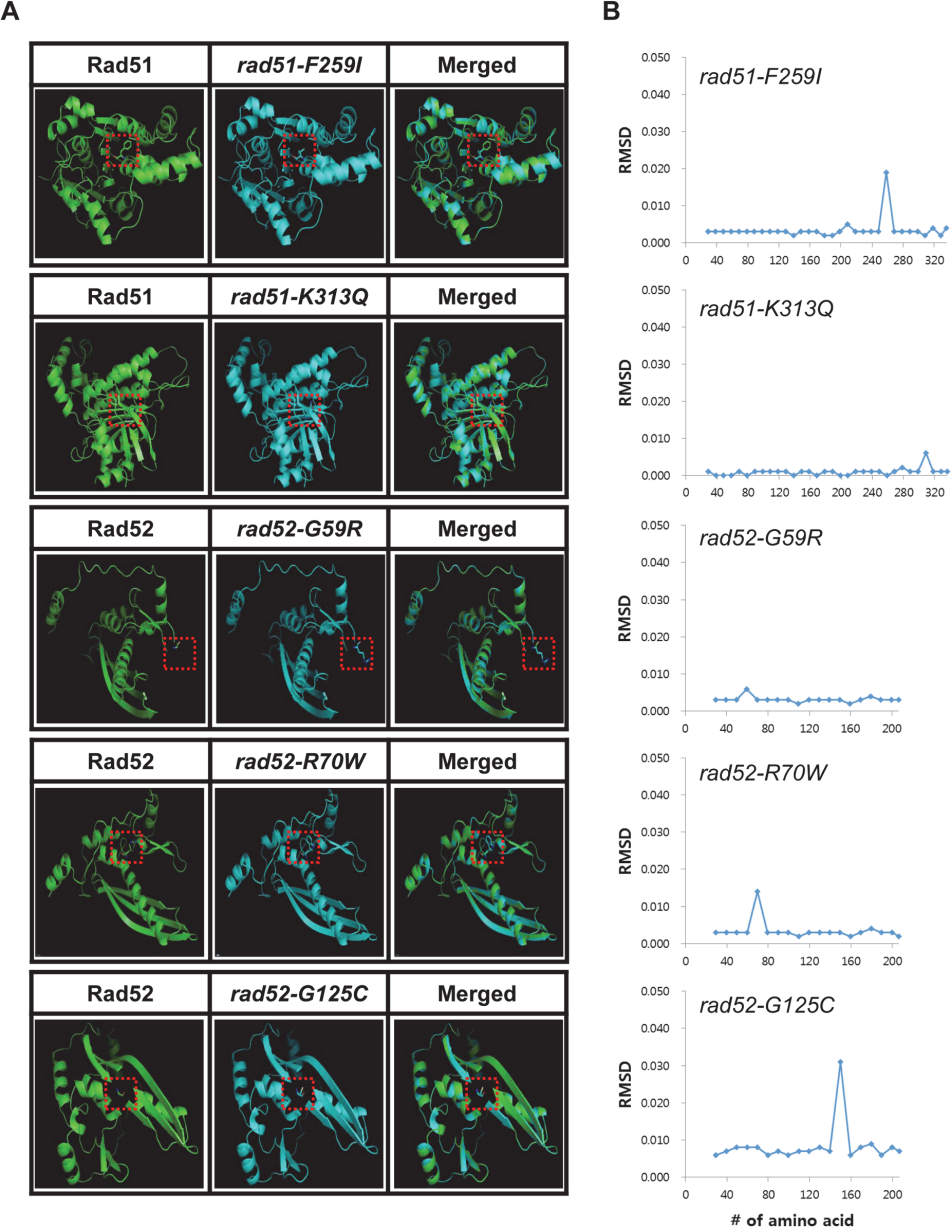
Protein structures of human wild-type proteins and the Rad51 and Rad52 variants. (A) Protein structures predicted by SWISS-MODEL were visualized by PyMOL. The wild type is represented in the left panel colored in green. The center panel shows the variants colored in aqua, and the right panel shows a merged structure. Variation sites were mapped by the full structure of amino acid side chains. Atoms of the side chains at the variation sites were coded by color (blue for nitrogen, red for oxygen, and yellow for sulfur). (B) Average Calpha RMSD of 10-amino acid fragments of variants of Rad51 and Rad52. Average RMSDs of 10 consecutive Calpha were plotted against their position. AUCs of RMSD were calculated by summation of the RMSD x 10. AUCpeaks (AUC of peaks) containing mutation sites were marked on the top of the peaks.

To determine whether the variants affected the structures of the functional domains, the WT and variant Rad51 and Rad52 proteins were analyzed with SWISS-MODEL (Fig 2A). Non-synonymous SNPs did not dramatically change the overall structure of the protein, but the RMSD changed for the regions of the amino acid replacements [33]. The impact of SNPs on the function of variant proteins can be predicted by measuring local RMSD, where the area under the curve (AUC) predicts functional variation (Fig 2B). The AUC of human *rad51-K313Q* was lower than that of human *rad51-F259I*. The AUC of human *rad52-G59R* was much lower than those of human *rad52-R70W* and *rad52-G125C*. However, functional assays for five mutant genes were not sufficient to determine the overall validity of this method for predicting functional loss due to point mutations. We are currently confirming the validity of the method using additional DNA repair genes in humans.

### Identification of the DNA Damage-sensitive Alleles of *RAD51* and *RAD52*

**Fig 3:**
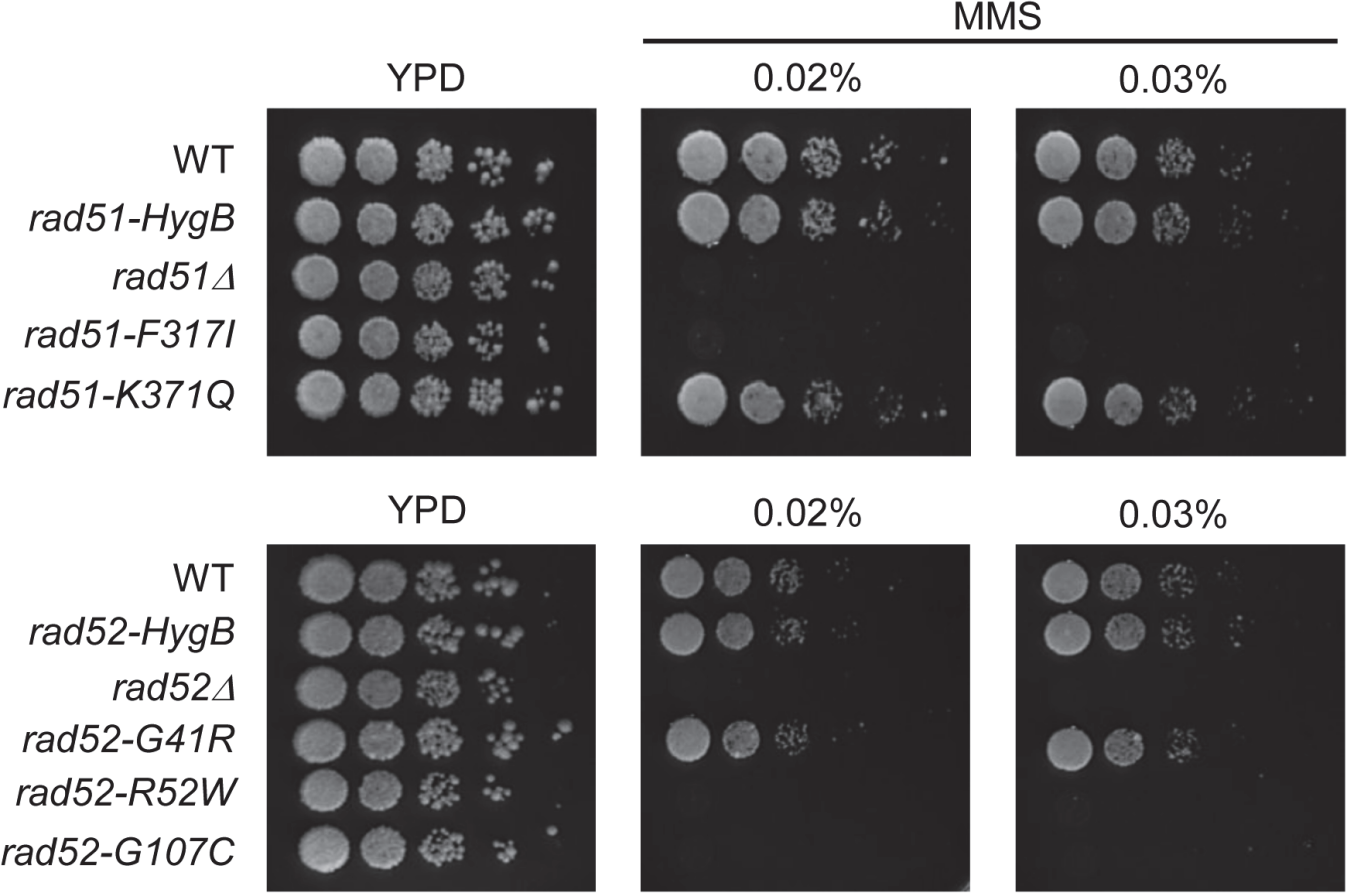
Sensitivity of *rad51* and *rad52* alleles to DNA damage. The spots from left to right represent 10-fold serial dilutions of the yeast cultures for WT, *rad51Δ*, *rad51-F317I*, *rad51-K371G*, *rad52Δ*, *rad52-G41R*, *rad52-R52W*, and *rad52-G107C* cells. Sensitivity to DNA damage was induced by methyl methane sulfonate (MMS). Cells were cultured in YPD liquid for 24 h, and then spotted onto YPD plates containing MMS (0.02% or 0.03%). These plates were incubated at 30°C.

To analyze whether the mutagenesis of human *RAD51* and *RAD52* had deleterious effects in the budding yeast system, we developed an *S*. *cerevisiae*-based assay system for the analysis of SNPs identified in human genes that are mutated at the same site in budding yeast (Figs 1 and 2). It has been reported that the absence of Rad51 or Rad52 leads to defects in DNA damage repair induced by MMS [34–38]. MMS-sensitive variants were selected to identify missense mutations that caused defects in HR as well as in other types of DNA damage responses (Fig 3). We observed that the variants *rad51* and *rad52* alleles were normally localized in the nucleus in DSB induced-damage conditions, as seen in the WT (S3 Fig). As shown in Fig 3, the *rad51-K371Q* mutant grew on the same MMS plate as the WT yeast. However, *rad51Δ* and *rad51-F317I* mutants demonstrated similar levels of MMS sensitivity. Because the *rad51-F317I* mutation site was located in the ATPase activity domain at the C-terminus, ATPase may not be functional in the presence of this mutation (Fig 3). These results are consistent with the conclusion that the ATPase activity of Rad51 is essential for its roles in DNA repair and HR [34, 35, 39]. Furthermore, the *rad52-R52W* and *rad52-G107C* strains were extremely sensitive to MMS, similar to *rad52Δ* (Fig 3). This result implied that the *rad52-R52W* and *rad52-G107C* mutations impaired the Rad52-dependent HR pathway. Because the mutation sites were in the DNA-binding domain, these mutants might have reduced DNA-binding activity. Moreover, we created the heterozygous strains expressing reduced levels of various *rad51* and *rad52* mutants to analyze MMS sensitivity of the variants accurately. The heterozygous diploid cells containing *RAD51/rad51-F317I*, *RAD52/rad52-R52W*, *and RAD52/rad52-G107C* could function normally in the repair of MMS-induced DNA damage (S4 Fig). In particular, we found that the heterozygous strains with *rad52-G41R* and other deleterious *rad52* alleles exhibited mild sensitivity to the same concentration of MMS (S4 Fig). Thus, we concluded that the *rad51-F317I*, *rad52-R52W*, and *rad52-G107C* sites have critical roles in DNA damage repair related to HR, and the complete loss-of-function mutations could not overcome DNA damage-sensitivity. In contrast to the prediction of PolyPhen and SIFT, *rad51-K371Q* and *rad52-G41R* activities related to DNA repair and damage responses were not completely suppressed. We further characterized the chromosome repair functions in physical analysis of DSB repair.

### Physical Analysis of Double-Strand Break Repair

**Fig 4:**
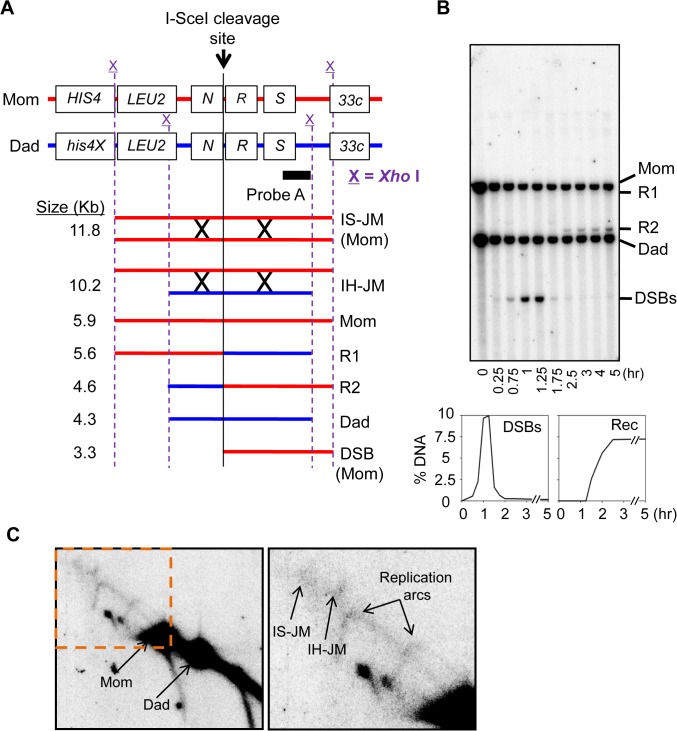
Physical analysis. (A) A map of the *HIS4LEU2* locus showing the diagnostic restriction endonuclease and selected probe-binding sites. *Xho*I restriction sites are indicated with an X. After addition of galactose, diploid cells expressed I*-Sce*I endonuclease from the Gal1 promoter generating double-strand breaks (DSBs) at the I-*Sce*I cleavage sites. R1, recombinant 1; R2, recombinant 2; IH-JM, interhomolog joint molecule; IS-JM, intersister joint molecule. (B) Representative image of a one-dimensional (1D) gel. Quantification of DSBs and recombinants. (C) Representative image of a two-dimensional (2D) gel. DNA species were detected by Southern blot hybridization using probe A.

To investigate mitotic DSB repair mediated by HR, we evaluated recombination intermediates and recombinants by one-dimensional (1D) and two-dimensional (2D) gel electrophoresis of the *HIS4LEU2* locus (Fig 4) [40]. Yeast cells were synchronized in the G1 phase and then transferred to YP media containing 2% galactose to induce I-*Sce*I enzyme expression. After I-*Sce*I induction, DSBs occurred at the I-*Sce*I cleavage site of the maternal chromosome at the *HIS4LEU2* locus (Fig 4A). I-*Sce*I induction of the Gal1 promoter was repressed by the addition of 3% glucose after a 45-min culture. Cell samples harvested at various time points were treated with psoralen-UV crosslinking and genomic DNA was purified as described previously [40–42]. *Xho*I-restriction fragments were separated by gel electrophoresis and hybridization signals identified by Southern blot analysis. DSBs and recombinants at individual time points were identified in 1D gel analysis and the levels were quantified (Fig 4B). DSBs within 3.3 kb of the site of I-*Sce*I cleavage in the *HIS4LEU2* locus of chromosome III were detected as a single band (Fig 4B). Furthermore, recombinants R1 (5.6 kb) and R2 (4.6 kb) were also detected in 1D gel analysis. To monitor the progress of recombination, 2D gel analysis of recombination intermediates was carried out to identify branched JMs, which are inter-strand DNA structures stabilized by the DNA crosslinking procedure (Fig 4C). JMs from native/native 2D gel analysis exhibited a distinct molecular weight and shape [40–42]. Because *Xho*I restriction polymorphisms were different for the maternal (“Mom”) *HIS4*::*LEU2* and paternal (“Dad”) *his4x*::*LEU2* loci, interhomolog JMs (IH-JMs), representing interhomolog interactions, could be distinguished from intersister JMs (IS-JMs), representing “Mom + Mom” interactions. We could identify the transient formation of IH-JMs and IS-JMs, in addition to replication Y-arc signals, in the 2D-gel analysis (Fig 4C).

### Recombinants Did Not Occur in Cells with *rad51-F317I*, *rad52-R52W*, and *rad52-G107C* Alleles

**Fig 5:**
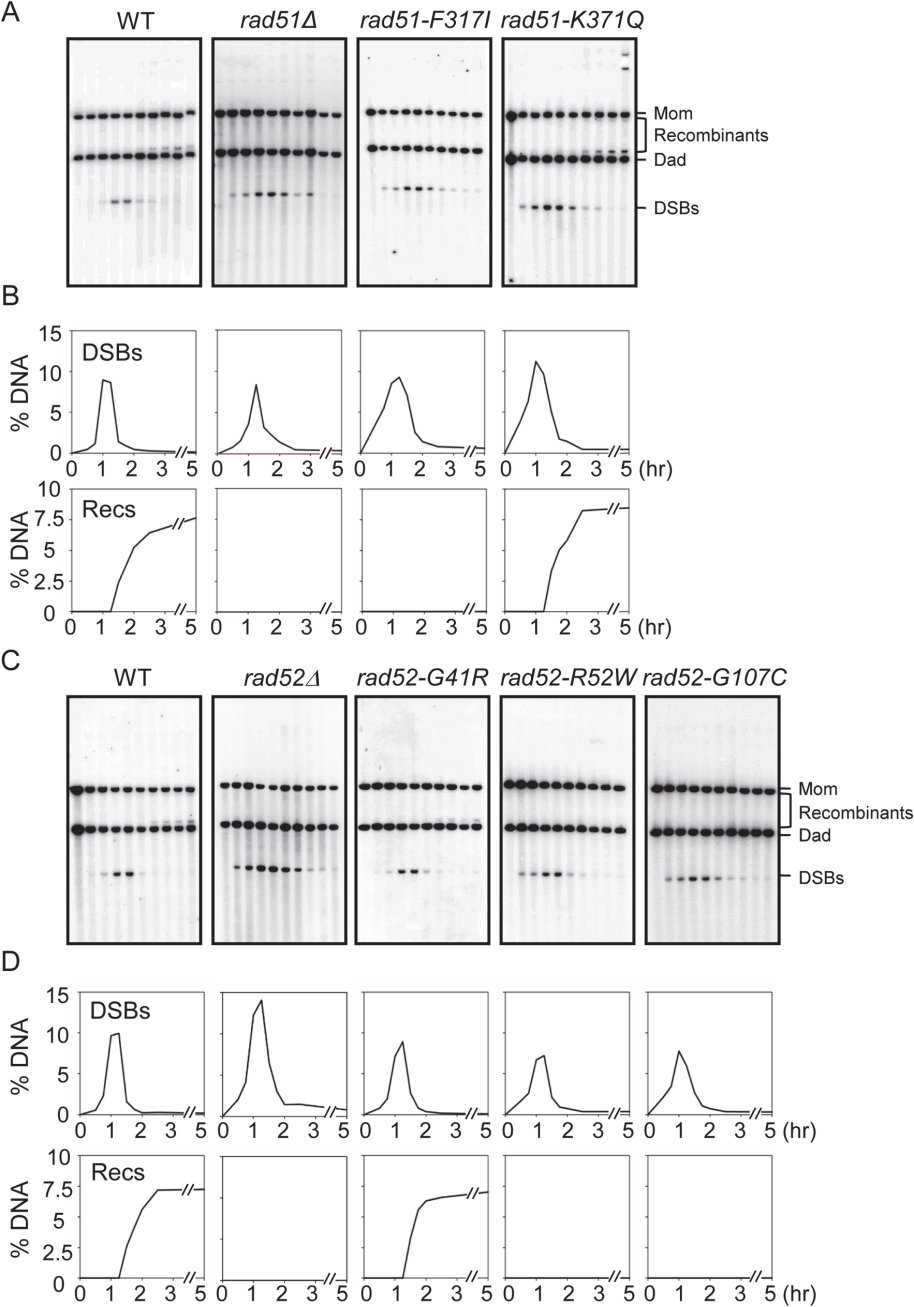
Analysis of the formation of DSBs and recombinants. (A and C) One-dimensional gel analysis of DSB and recombinant formation over the time course in WT (KKY940), *rad51Δ* (KKY1088), *rad51-F317I* (KKY1086), *rad51-K371Q* (KKY1091), *rad52Δ* (KKY1978), *rad52-G41R* (KKY1143), *rad52-R52W* (KKY1145), and *rad52-G107C* (KKY655) cells. (B and D) Quantification of DSBs and recombinants. DSB, double strand break; Rec, recombinant.

In the mitotic cell cycle of diploid cells, IH-JMs are formed and then resolved into IH-recombinants [40]. To investigate the function of *rad51* and *rad52* alleles in DSB repair, we prepared a galactose inducible I-*Sce*I system as described above. In the WT strain, DSBs were first detected 30 min after the addition of galactose to the YP medium; they peaked at 1h, and were processed efficiently to produce recombinants (Fig 5A and 5B). The timing of DSBs occurrence in *rad51-F317I*, *rad51-K371Q*, *rad52-G41R*, *rad52-R52W*, and *rad52-G107C* mutants were similar to those in the WT strain (Figs 5 and S5); DSBs were detected at 15 min and then gradually decreased, but small quantities of DSBs remained up to 5 h. Thus, all strains produced proper DSBs and efficiently processed DSB repair. However, the DSB life spans differed slightly among strains owing to factors such as among-culture differences in cell synchronization, Southern hybridization, and quantification of signals (Figs 5 and S5). In the WT strain, recombinants also appeared to form, and plateaued at approximately 7.5% (Fig 5B and 5D). As shown in WT, recombinants were observed in *rad51-K371Q* and *rad52-G41R*, suggesting that these variants process DSB repair via the HR pathway in a normal manner. However, recombinants were not detected in the *rad51-F317I*, *rad52-R52W*, and *rad52-G107C* mutant strains, as observed in *rad51Δ* and *rad52Δ* mutant cells. Consistent with the results from MMS analysis of the mutant strains, *rad51-F317I*, *rad52-R52W*, and *rad52-G107C* cells exhibited inefficient DSB repair-mediated recombination (Figs 3 and 5).

### JM Formation Was Abolished in Deleterious *rad51* and *rad52* Mutations

**Fig 6:**
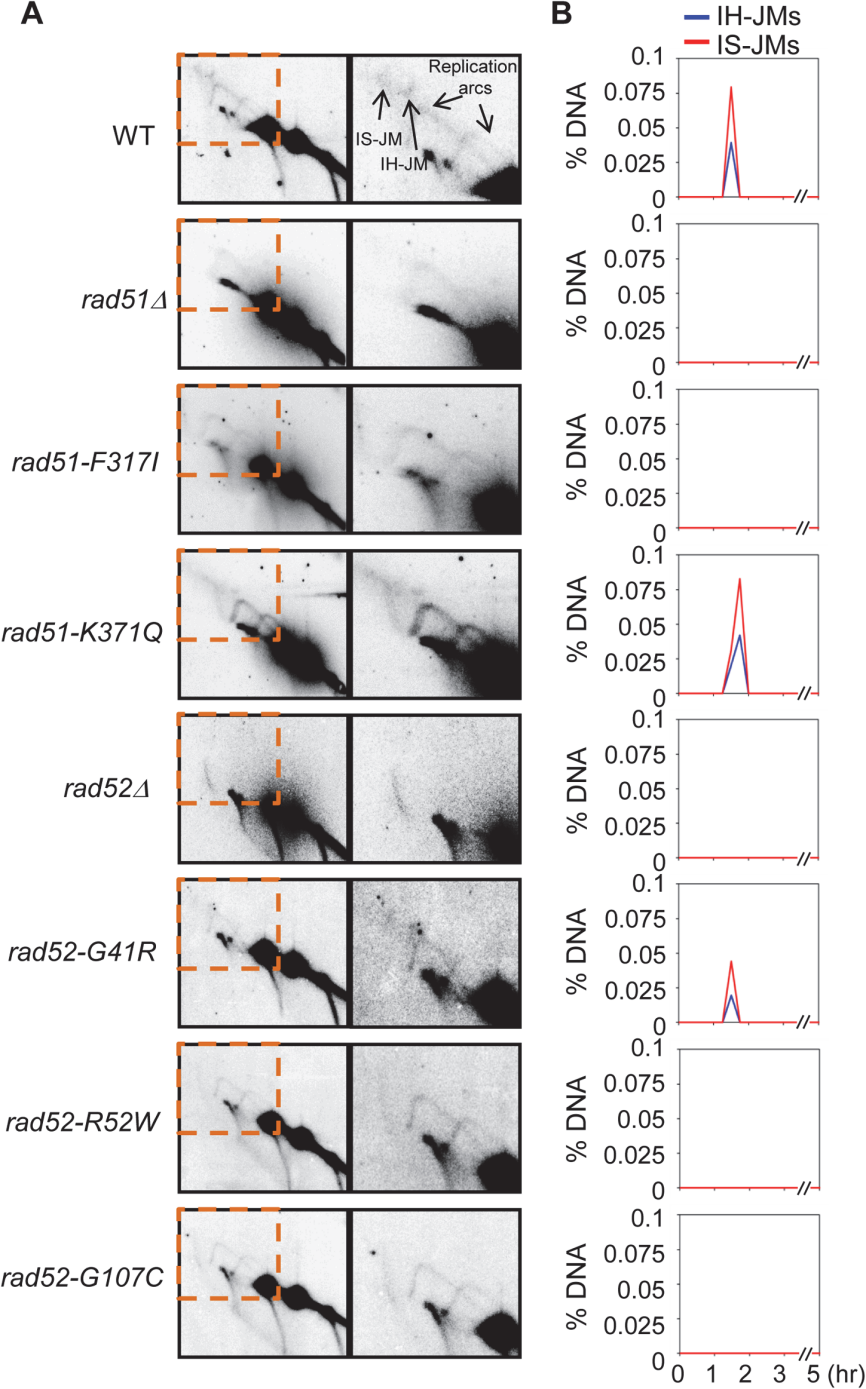
Analysis of JMs during DSB repair. (A) Two-dimensional gel of JM formation over the time course in WT (KKY940), *rad51Δ* (KKY1088), *rad51-F317I* (KKY1086), *rad51-K371Q* (KKY1091), *rad52Δ* (KKY1978), *rad52-G41R* (KKY1143), *rad52-R52W* (KKY1145), and *rad52-G107C* (KKY655) cells. (B) Quantification of JM formation. JM, joint molecule.

The structure of mitotic recombination intermediates was further investigated with native/native 2D gel analysis in which the DNA fragments were separated based on molecular weight in the first dimension and shape in the second dimension. In all experiments, cellular DNA was subjected to psoralen-UV crosslinking prior to DNA isolation immediately after harvesting cells from each time point. Psoralen-UV crosslinking stabilizes recombination-associated JMs such as single-end invasions (SEIs) and double-Holliday junctions (double-HJs). After DNA replication, “Mom + Mom” IS-JM species, representing sister chromatid interactions established for the repair of DSBs, were expected to form a JM of 11.8 kb (Fig 4A and 4C). “Mom + Dad” IH-JM species, which represented recombinant intermediates, were expected to form a JM of 10.2 kb (Fig 4A and 4C). Thus, high levels of IH-JM and recombinant intermediate formation represented efficient interhomolog interactions between “Mom” and “Dad” chromosomes. The double-HJ was presupposed to be the major intermediate that has been identified in the progress of meiotic recombination [40]. Moreover, using native/denaturing 2D gel analysis, Hunter and colleagues found that double-HJs are intermediates of DSB-promoted recombination in mitotic cells and that a portion of IH-JMs could also be recombinant single-HJs [40]. Thus, the IH-JMs observed in this study could also include both double-HJs and single-HJs. DNA replication arcs in all strains appeared at a similar time, indicating that the mutant alleles did not affect the progression of DNA replication. The JM species were analyzed sequentially on Southern blots with a specific probe that bound to either the “Mom” or “Dad” homolog (Fig 6A). WT cells formed approximately 0.07% IS-JM (“Mom + Mom”) and approximately 0.03% IH-JM (“Mom + Dad”) at 1.75 h after addition of galactose to the cultures (Fig 6B). In this assay system, “Dad + Dad” IS-JMs could not form because DSBs only occurred at the I-*Sce*I site of the “Mom” chromosome. In *rad51-K371Q* cells, the spectrum of JM is similar to that of WT cells. Interestingly, *rad52-G41R* cells appear to form slightly lower JM levels than WT, which suggests that the variant was partially defective in JM formation, and additional evidence confirmed this, i.e., the MMS-sensitivity test for heterozygous strains with *rad52Δ* or *rad52* deleterious alleles (Figs 6B and S4 and S6). However, both IH-JMs and IS-JMs were not detected in 2D gel analysis of *rad51-F317I*, *rad52-R52W*, and *rad52-G107C* cells (Figs 6 and S6). The *rad51-F317I*, *rad52-R52W*, and *rad52-G107C* mutations resulted in cells that were severely deficient in JM formation and could not form recombinant products (Figs 5 and 6). These results suggested that *rad51-F317I*, *rad52-R52W*, and *rad52-G107C* mutations impaired the strand exchange activity of the HR pathway and led us to conclude that the three mutations play a crucial role in the DSBR pathway. However, DSBs in the mutant strains were largely disappeared over a 5-h period. We inferred that DSBs in the strains carrying mutant alleles were most likely processed by a synthesis-dependent strand annealing (SDSA) mechanism, which yields non-crossovers or disappears by DSB-end hyperresection.

### Deleterious *rad51* and *rad52* Alleles Affect the Efficiency of Sporulation

**Fig 7:**
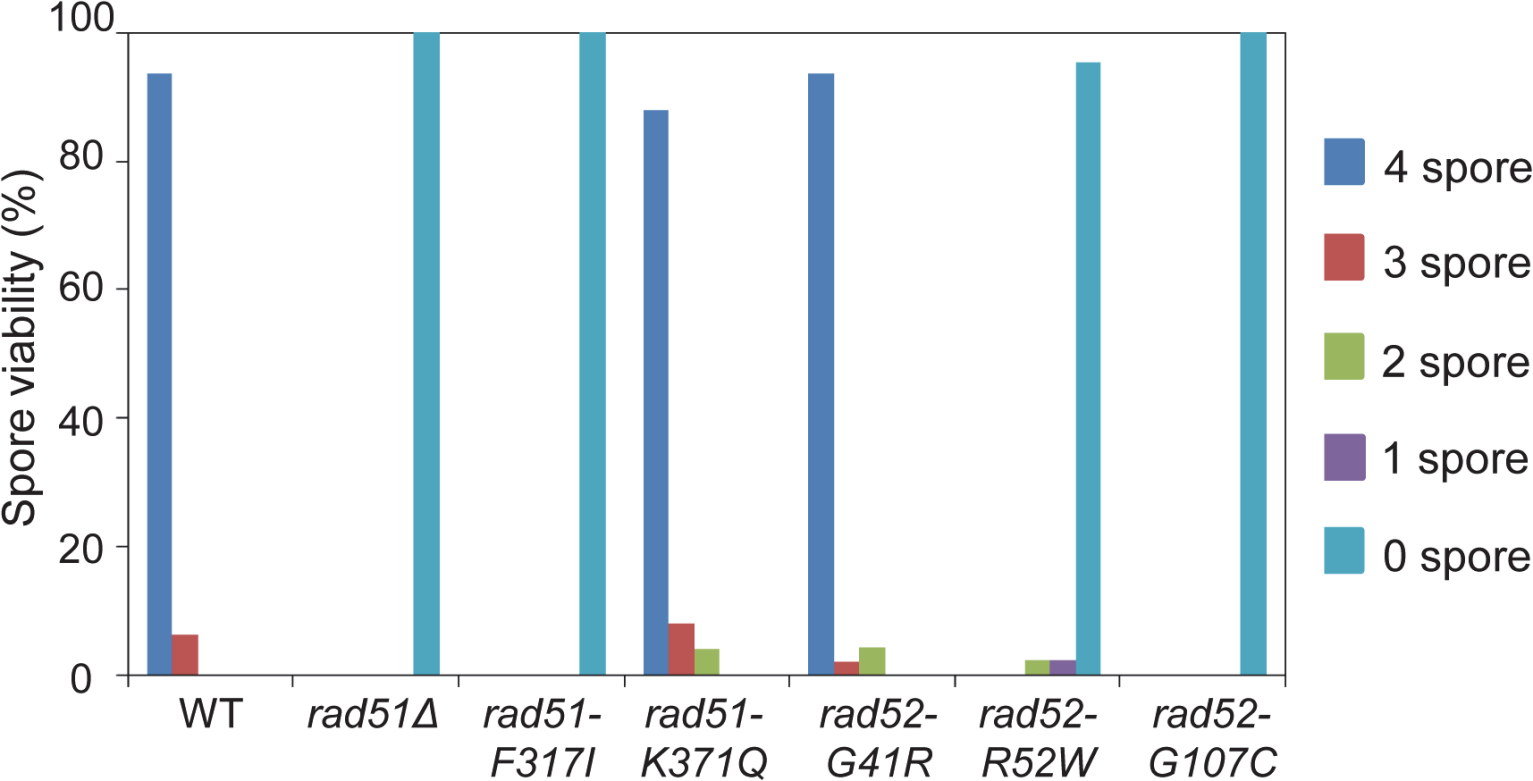
Spore viability of Rad51 and Rad52 variants. Analysis of meiotic spore viability in parallel cultures for WT, *rad51*, and *rad52* mutant strains. Cells were cultured in SPM (1% potassium acetate and 0.02% raffinose) for 18 h to induce sporulation. Approximately 100 tetrads were dissected for each strain.

During meiosis, yeast cells with unsegregated DNA fail to form mature spores. We investigated whether deleterious *rad51* and *rad52* alleles affect the efficiency of spore formation. The *rad51* and *rad52* alleles were cultured to induce sporulation in SPM media for 24 h at 30°C, and spore viability was analyzed after tetrad dissection. In WT cell, 94% of tetrads underwent normal sporulation based on the levels of viable spores, however, the spores of *rad51Δ*, *rad51-F317I*, *rad52-R52W*, and *rad52-G107C* cells were not viable, in accordance with the physical DNA analysis results (Fig 7). The *rad51-K371Q* and *rad52-G41R* mutations gave rise to 88 and 94% viable spores, respectively (Fig 7). This result further suggested that the deleterious SNPs (*rad51-F317I*, *rad52-R52W*, and *rad52-G107C*) inactivated the DSBR pathway and prevented formation of viable spores.

## Conclusion

HR is a fundamental mechanism for the repair of DNA DSBs caused by ionizing radiation, ultraviolet light, chemical agents, and replication errors. Mitotic HR supports genomic stability and suppresses tumorigenesis via the proper repair of DSBs [43]. Mitotic crossing-over is a rare but important phenomenon. In human cancer, it is thought to play a major role in enabling the expression of recessive cancer-causing mutations [44]. Previous studies have shown that Rad51 can interact with the single-stranded DNA-binding proteins RPA and Rad52, and catalyzes the ATP-dependent strand exchange between homologous sequences [45]. The Rad52 DNA-binding site is critical for the establishment of heteroduplex joints required in the formation of the D-loop by the Rad51-Rad52 complex, and this complex function may be important for homologous recombination as well [46]. Rad54 is also a key player in HR and physically interacts with Rad51 to perform the homology search and strand invasion steps in HR [47]. The mammalian homolog to yeast Rad54, *Rad54B*, has been identified in human cells and encodes a protein that consists of 747 amino acids and is 52% identical to yeast Rad54 [48, 49]. However, in this study, we could not identify *rad54* alleles as causal variants because the sequence similarity of the functional domain was low between human and yeast.

Systematic approaches using *S*. *cerevisiae* alleles to examine mammalian genes have been well established and successfully used for screening mutants affecting DNA damage repair. In this study, the approach included bioinformatic and structure-based predictions were used to identify missense mutations in human *RAD51* and *RAD52* genes. We identified a number of deleterious alleles of the human Rad51 and Rad52 proteins that were defective in the strand exchange activity of HR using a yeast-based system. Based on the *in vivo* analysis, we estimated that the accuracy of functional variation prediction by SIFT and PolyPhen was 60%, which is impressive compared to previous estimates [50, 51]. Furthermore, protein structural analysis of Rad51 and Rad52 variants showed that the mutation sites of the deleterious alleles were functionally important for the mediation of DSB repair (Fig 2). Through the physical analysis of recombination intermediates, we observed that the formation of JMs and recombinants was abolished in *rad51-F317I*, *rad52-R52W*, and *rad52-G107C* mutants, similar to *rad51* or *rad52* deletion mutants. These results suggested that the ATPase activity domain of Rad51 and the DNA-binding domain of Rad52 are crucial for DNA repair and that mutations in these regions might be associated with various cancers.

Model systems for the analysis of DNA damage repair have the potential to be used for identifying and understanding novel genes and pathways relevant to human cancer. Further studies in human patients with various cancer types may support these new insights into potential disease mechanisms and therapeutic strategies. We anticipate that the deleterious mutations of human HR genes verified herein could contribute to genomic instability and be related to cancer initiation and progression.

## Materials and Methods

### Single Nucleotide Polymorphism Selection

Single nucleotide polymorphisms (SNPs) in *RAD51* and *RAD52* were selected from the National Center for Biotechnology Information Single Nucleotide Polymorphism (NCBI SNP) database using the following methods. (i) Human *RAD51* and *RAD52* gene sequences were compared to corresponding sequences in other species, including yeast. (ii) SNPs in the functional domain were selected. (iii) The selected human and yeast SNPs were matched by position. (iv) Finally, the impact of the amino acid substitutions was predicted using Sorting Intolerant From Tolerant (SIFT) and Polymorphism Phenotyping (PolyPhen) to select “damaging” and “deleterious” SNPs. The open reading frames (ORFs) of *RAD51* and *RAD52* were aligned by EBI ClustalW (http://www.ebi.ac.uk/Tools/msa/clustalw2/). SNPs were selected from the NCBI SNP database (http://www.ncbi.nlm.nih.gov/snp/) and confirmed to be located in exons. The *RAD51* and *RAD52* SNPs were processed using SIFT and PolyPhen. SNPs located in exons were examined using SIFT and PolyPhen (http://asia.ensembl.org/Homo_sapiens/Gene/) to select deleterious and damaging SNPs.

### Yeast Strains

SK1 strains were used in this study and are described in S1 Table. The *HIS4LEU2* locus and I-*Sce*I cleavage site have been described previously [40].

### Construction of Plasmids and Mutagenesis of *RAD51* and *RAD52*

The *RAD51* and *RAD52* open reading frames (ORFs) were amplified by polymerase chain reaction (PCR) using primers flanking the ORFs and containing restriction sites to produce *Hind*III and *Pac*I sites. PCR products were digested with *Hind*III and *Pac*I and cloned into the corresponding sites of pFA6a-NT1 plasmid vectors. To create *rad51* and *rad52* mutations, the ORFs and selection marker regions were amplified using the pairs of primers (shown in S1 Table). Forward primers included a point mutation at the 3' end and reverse primers included the homologous yeast sequence. The *RAD51* primers were generated as follows (mutant bases are underlined): *rad51-F317I* forward, 5'-CTTTGCAAAGGCTGGCCGACCAAATT-3'; *rad51-K371Q* forward, 5'-GGTAAGGGATGTCAAAGATTATGCCAA-3'; and reverse primer, 5'-GAAAAAGAGGAGAATTGAAAGTAAACCTGTGTAAATAAATAGAGACAAGAGACCAAATAC GTTAAAGCCTTCGAGCGACCC-3'. *RAD52* primers were generated as follows (mutant bases are underlined): *rad52-G41R* forward, 5'-ATATCTCCAAGAGAGTTGGGTTTAGA-3'; *rad52-R52W* forward, 5'-GATTGCATACATCGAAGGTTGGTGG-3'; *rad52-G107C* forward, 5'-CGGGACTTATAGGGAAGATATTTGT-3'; and reverse primer, 5'-AGGATTTTGGAGTAATAAATAATGATGCAAATTTTTTATTTGTTTCGGCCAGGAAGCGTTGTTAAAGCCTTCGAGCGTCCCAAAA-3'. PCR products were transformed into the SK1 strain and the cells were then grown on hygromycin B (200 mg/L) plates for 3 days at 30°C. The *RAD51* and *RAD52* ORFs from single colonies grown on hygromycin B plates were confirmed by DNA sequencing using the ABI Prism BioDye Terminator version 3.1.

### Mitotic Time Course

Diploid cells were cultured in YPD (1% bacto yeast extract, 2% bacto peptone, and 2% glucose) for 24 hr. To synchronize cells in the G1 phase, the YPD cultures were diluted 1:500 and grown in SPS (0.5% bacto yeast extract, 1% bacto peptone, 0.67% yeast nitrogen base, 1% potassium acetate, and 50 mM potassium biphthalate, pH 5.5) for 18 hr at 30°C. Synchronized cells were then washed once and resuspended with fresh YP including 2% galactose to induce I-*Sce*I expression. After 45 min, 3% glucose was added to repress I-*Sce*I expression. Cells were harvested at the time points.

### DNA Physical Analysis

Genomic DNA preparation and DNA physical analysis were performed as described previously [42, 52–54]. Cells were cross-linked prior to spheroplasting by exposing them to 360 nm UV light in the presence of 0.1 mg/mL trioxsalen (Sigma, St. Louis, MO, USA). Cells were then spheroplasted, and the DNA was extracted as described [42]. For the physical analysis of recombination in 2-dimensional (2D) gels, 4 μg of genomic DNA was digested with *Xho*I and precipitated in > 99% ethanol. Digested DNA samples were allowed to separate in 0.6% agarose gel in TBE buffer (89 mM Tris-borate and 2 mM EDTA, pH 8.3) without ethidium bromide at 2 V/cm for 24 hr. Gels were stained with ethidium bromide, and the lanes were cut out and placed in a 2D gel tray. Electrophoresis in the second dimension was carried out at 5 V/cm for 6 hr. Gels were subjected to Southern hybridization analysis using Probe A (Fig 3). Quantification of DNA species was performed using a phosphoimage analyzer and the Quantity One software (Bio-Rad, Hercules, CA, USA). The cell cycle was monitored by fluorescent-activated cell-sorting using Sytox Green as described previously [55].

### Methyl Methane Sulfonate Test

Cells were grown to saturation in YPD at 30°C overnight and were serially diluted by 10-fold decreases (10^–1^, 10^–2^, 10^–3^, 10^–4^, and 10^–5^ cells), and spotted onto YPD plates containing methyl methane sulfonate (MMS; 0.02% or 0.03%). After incubation at 30°C for 3 days, the plating efficiency and cell growth were evaluated.

### Immunofluorescence Analysis

Yeast cells were fixed with 3% paraformaldehyde in PBS for 20 min. Then, the cells were treated with 0.3% Triton X-100 in PBS, and rinsed with PBS. An anti-Rad51 (Cat. No. sc-33626) or anti-Rad52 (Cat. No. sc-50445) polyclonal antibody were used for immunostaing. The following day, the cells were washed five times for 5 min each time with 2% BSA in PBS, then incubated with FITC-conjugated anti-rabbit IgG (H+L) secondary antibody (Jackson ImmunoResearch, West Grove, PA, USA) at a dilution of 1:200 for 1 h at room temperature. Cells were washed three times with blocking buffer, and then rinsed with PBS at room temperature. The nuclei were incubated with DAPI and mounted using Dako fluorescent mounting medium (Dako Corporation, Carpenteria, CA, USA). Immunofluorescence was visualized on an Olympus BX53 Fluorescence Microscopy System (Olympus, Tokyo, Japan).

### Prediction of Structural Changes in the Variants

Structural alterations of Rad51 and Rad52 at the protein level induced by SNPs were determined by SWISS-MODEL (http://swissmodel.expasy.org/) and visualized by PyMOL (The PyMOL Molecular Graphics System, Version 0.99 rc6 Schrödinger, LLC., New York, NY, USA). Overall structural changes induced by side chain variations could be measured by comparisons of the root-mean-square deviation (RMSD) of C-alpha. The overall RMSD reflects the average distance between all of the backbone atoms of wild type (WT) and variant proteins. The RMSDs of Calphas of consecutive 10-amino acid-long fragments (RMSD_10aa_) were measured using PyMOL.

## Supporting Information

S1 Fig Construction of Rad51 and Rad52 variants using PCR. The SNP regions of RAD51 or RAD52 were amplified using the pairs of primers (see Materials and Methods). The arrows indicate primer-binding sites. PCR products were transformed into yeast cells, and then cells were grown on hygromycin B plates.
(TIF)

S2 Fig Amino acid sequence alignment of Rad51 and Rad52 in various species. Rad51 and Rad52 sequences are aligned in various species, including *H*. *sapiens and S*. *cerevisiae*, *P*. *troglodytes*, *C*. *lupus*, *B*. *taurus*, *R*. *norvegicus*, *M*. *musculus*, *G*. *gallus*, *D*. *rerio*, *K*. *lactis*, *E*. *gossypii* by use of clastalW program. Red box, selected SNPs for this study.
(TIF)

S3 Fig Nuclear localization of Rad51 and Rad52 in response to DNA damage. Cells were incubated with 0.1% MMS and subjected to immunofluorescence as described in Materials and Methods. WT, *rad51-F317*, *rasd51-K371Q*, *rad52-G41R*, *rad52-R52W*, and *rad52-G107* cells were stained with rabbit anti-Rad51 or anti-Rad52 polyclonal antibody, followed by staining with anti-IgG conjugated with FITC. DNA was stained with DAPI.
(TIF)

S4 Fig MMS sensitivity of the heterozygous strains containing various *rad51* and *rad52* alleles. Sensitivity to DNA damage was induced by methyl methane sulfonate (MMS). Cells were cultured in YPD liquid for 24 h, and then spotted onto YPD plates containing 0.02% MMS.
(TIF)

S5 Fig Analysis of DSB and recombinant formation. (A and C) One-dimensional gel analysis of DSB and recombinants formation over the time course in *WT* (KKY940), *rad51Δ* (KKY1089), *rad51-F317I* (KKY1086), *rad51-K371Q* (KKY1091), *rad52-G41R* (KKY1143), *rad52-R52W* (KKY1145), and *rad52-G107C* (KKY655). (B and D) Quantification of DSBs and recombinants.
(TIF)

S6 Fig Analysis of JMs during DSB repair. (A) Two-dimensional gel of JM formation over the time course in WT (KKY940), *rad51Δ* (KKY1088), *rad51-F317I* (KKY1086), *rad51-K371Q* (KKY1091), *rad52Δ* (KKY1142), *rad52-G41R* (KKY1143), *rad52-R52W* (KKY1145), *rad52-G107C* (KKY655). (B) Quantification of JM formation.
(TIF)

S1 Table Strain list. (DOCX)

S2 Table Non-synonymous SNPs in functional domains of human *RAD51* and *RAD52*. (DOCX)
