# Supplementary figures and images for: Functional Validation of Rare Human Genetic Variants Involved in Homologous Recombination Using Saccharomyces cerevisiae
Source: PLoS One. 2015 May 4;10(5):e0124152. doi: 10.1371/journal.pone.0124152 (PMC4418691; doi:10.1371/journal.pone.0124152)

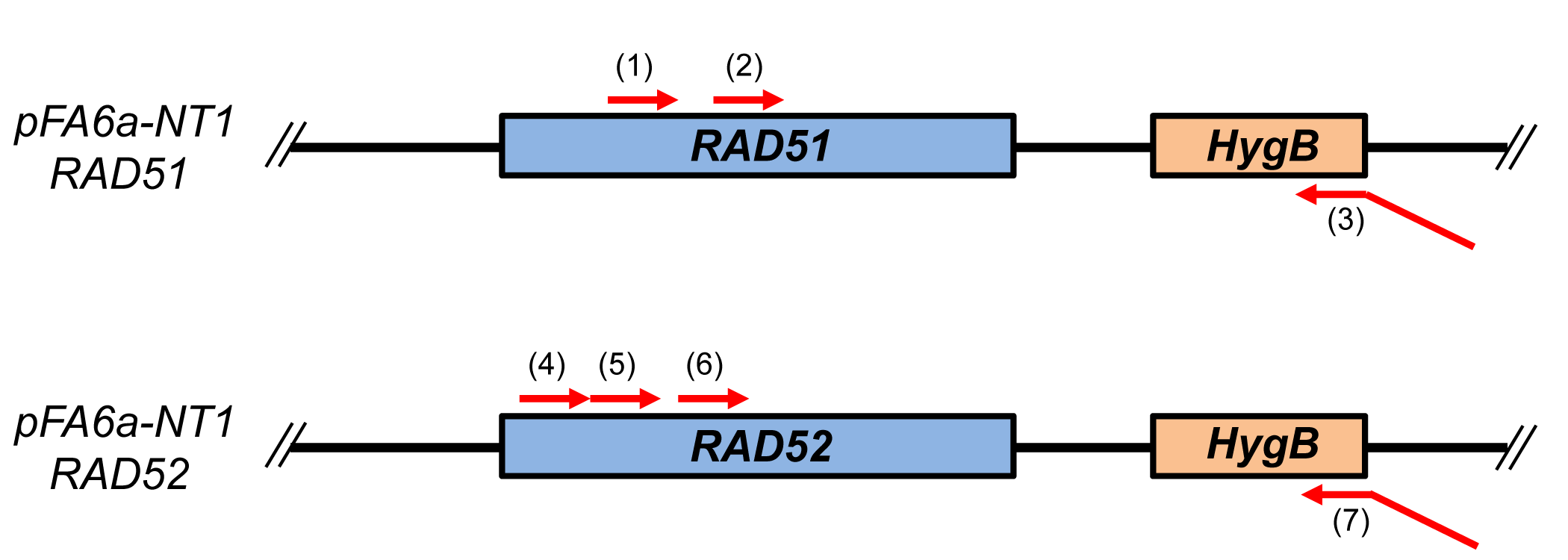

Supplement: S1 Fig — The SNP regions of RAD51 or RAD52 were amplified using the pairs of primers (see Materials and Methods). The arrows indicate primer-binding sites. PCR products were transformed into yeast cells, and then cells were grown on hygromycin B plates. (TIF) [file pone.0124152.s001.tif]

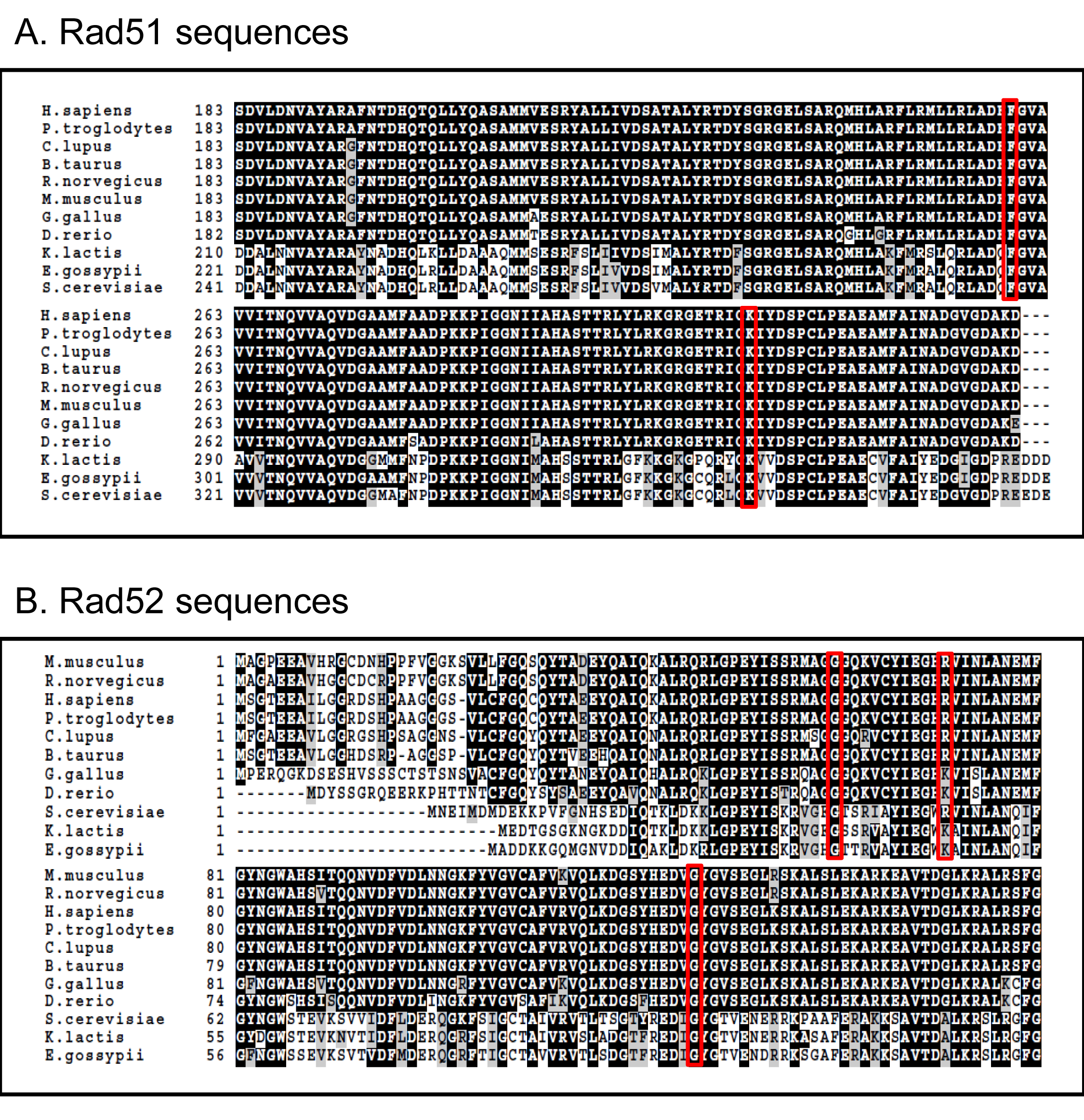

Supplement: S2 Fig — Rad51 and Rad52 sequences are aligned in various species, including H. sapiens and S. cerevisiae, P. troglodytes, C. lupus, B. taurus, R. norvegicus, M. musculus, G. gallus, D. rerio, K. lactis, E. gossypii by use of clastalW program. Red box, selected SNPs for this study. (TIF) [file pone.0124152.s002.tif]

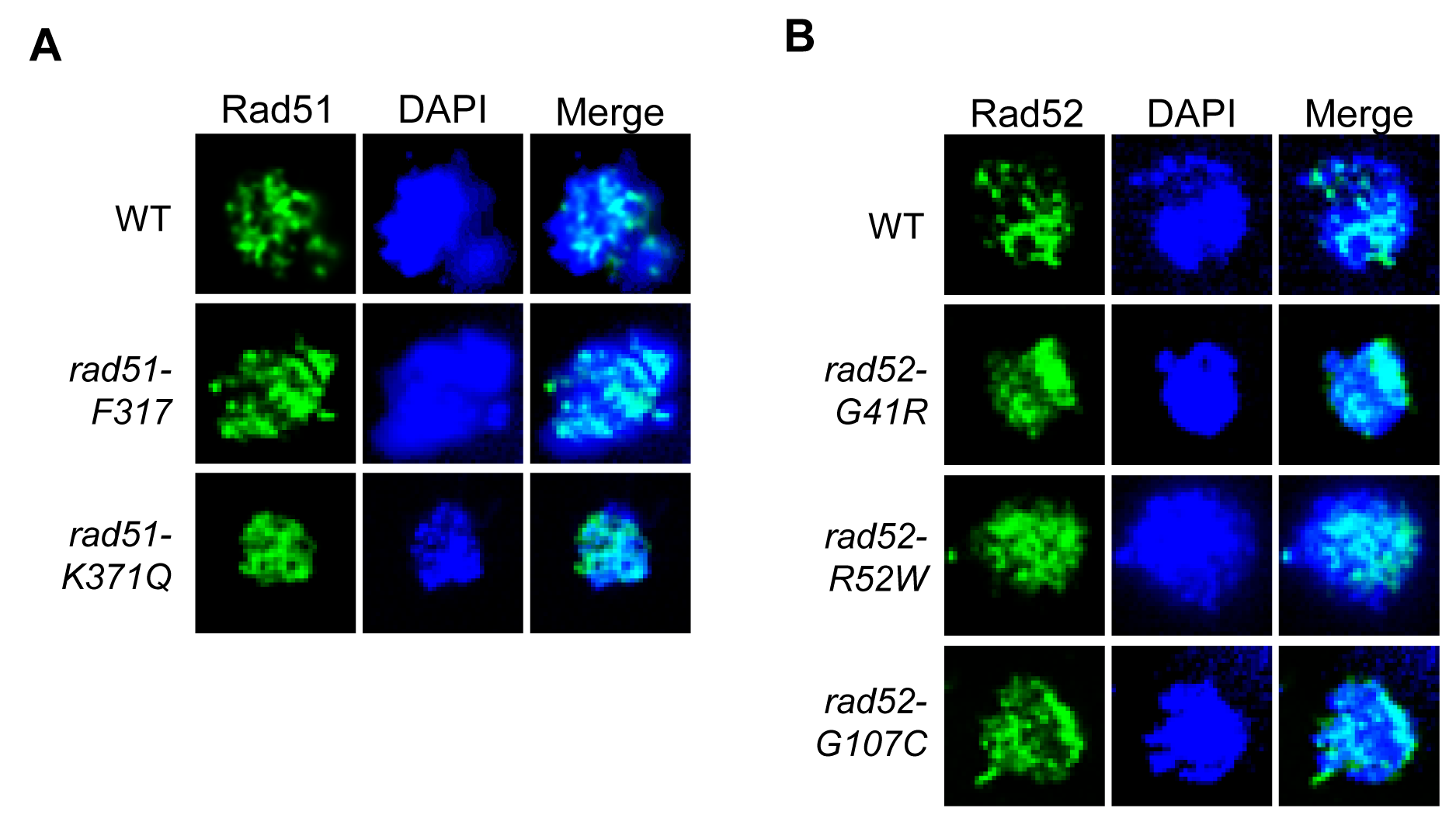

Supplement: S3 Fig — Cells were incubated with 0.1% MMS and subjected to immunofluorescence as described in Materials and Methods. WT, rad51-F317, rasd51-K371Q, rad52-G41R, rad52-R52W, and rad52-G107 cells were stained with rabbit anti-Rad51 or anti-Rad52 polyclonal antibody, followed by staining with anti-IgG conjugated with FITC. DNA was stained with DAPI. (TIF) [file pone.0124152.s003.tif]

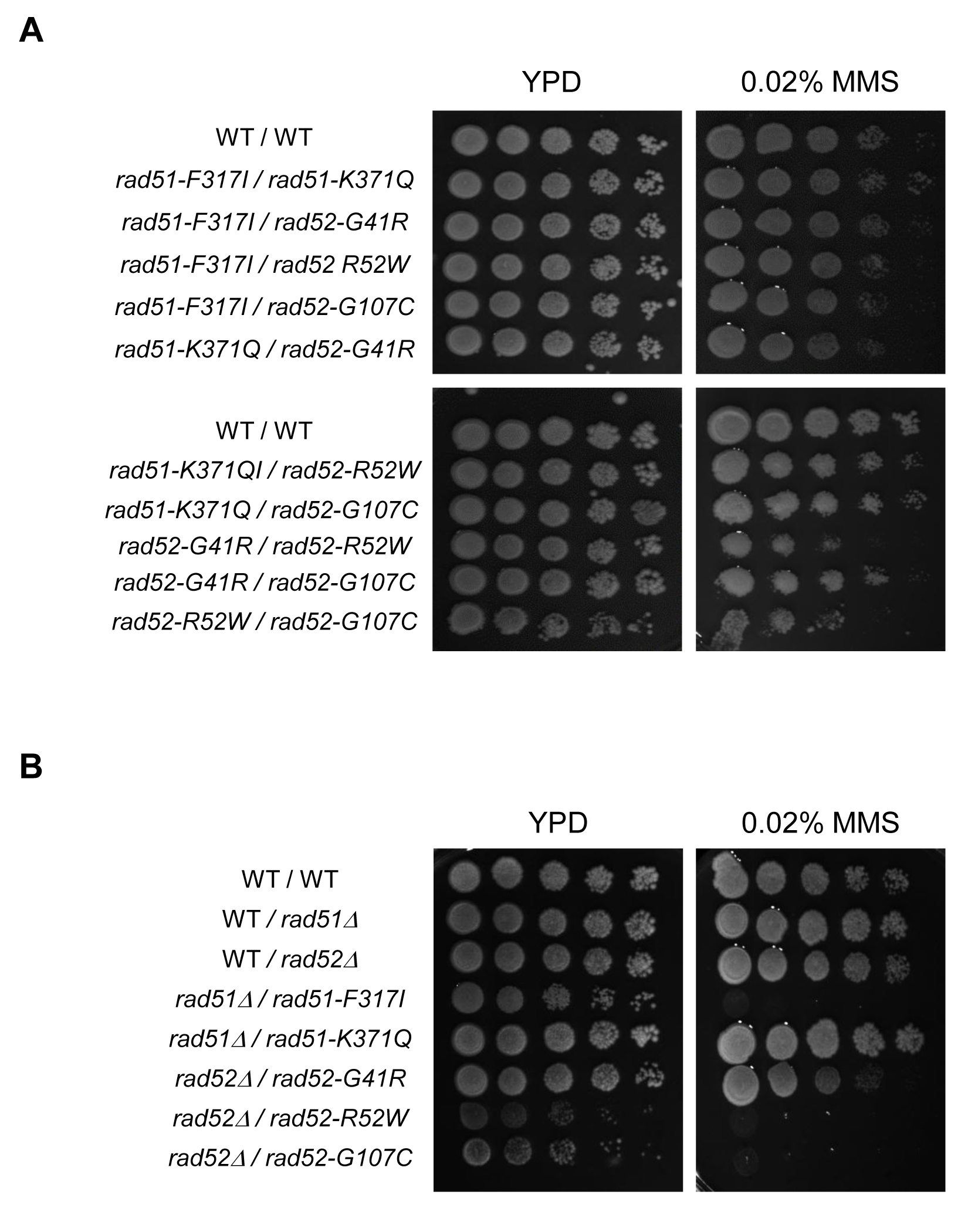

Supplement: S4 Fig — Sensitivity to DNA damage was induced by methyl methane sulfonate (MMS). Cells were cultured in YPD liquid for 24 h, and then spotted onto YPD plates containing 0.02% MMS. (TIF) [file pone.0124152.s004.tif]

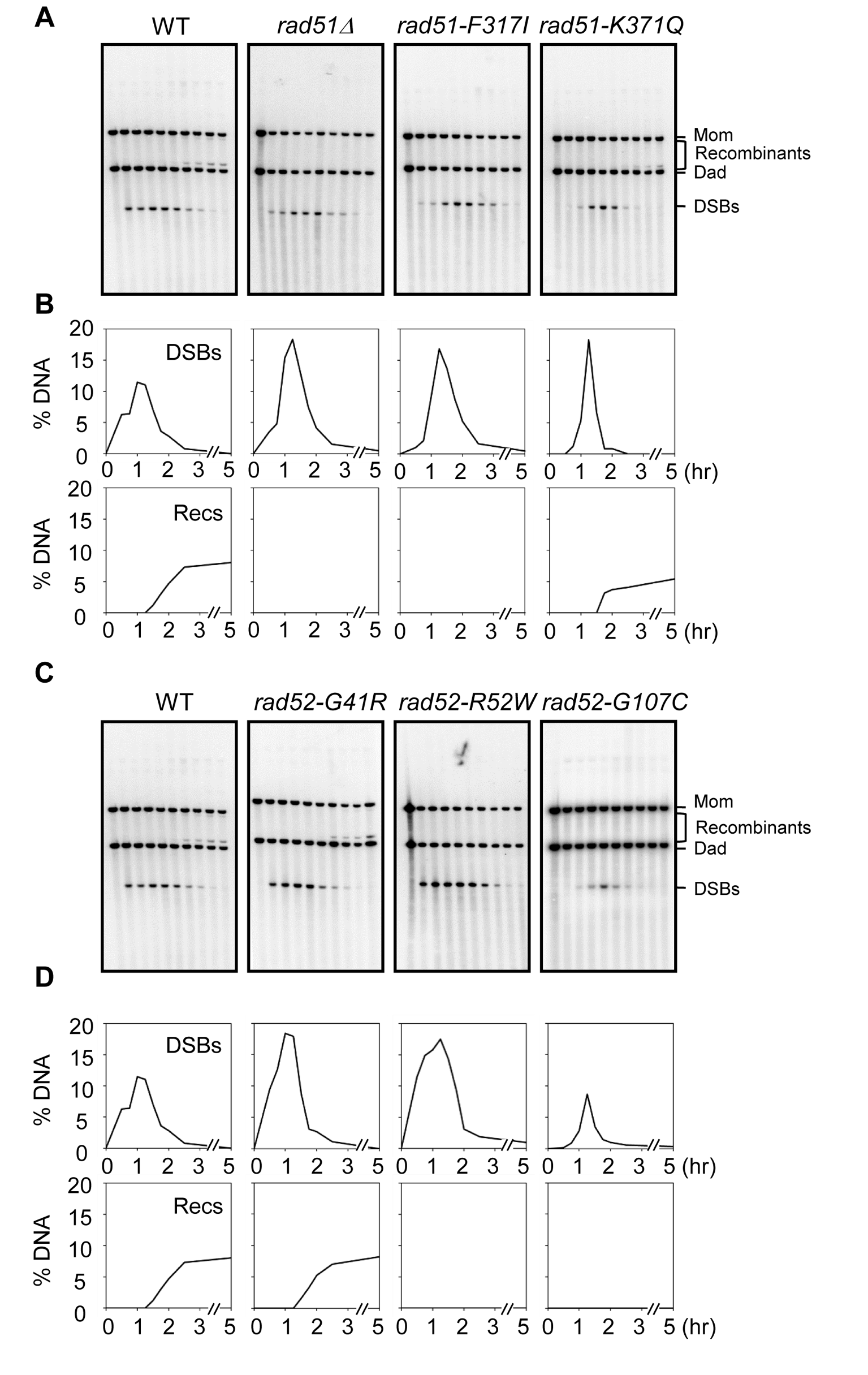

Supplement: S5 Fig — (A and C) One-dimensional gel analysis of DSB and recombinants formation over the time course in WT (KKY940), rad51Δ (KKY1089), rad51-F317I (KKY1086), rad51-K371Q (KKY1091), rad52-G41R (KKY1143), rad52-R52W (KKY1145), and rad52-G107C (KKY655). (B and D) Quantification of DSBs and recombinants. (TIF) [file pone.0124152.s005.tif]

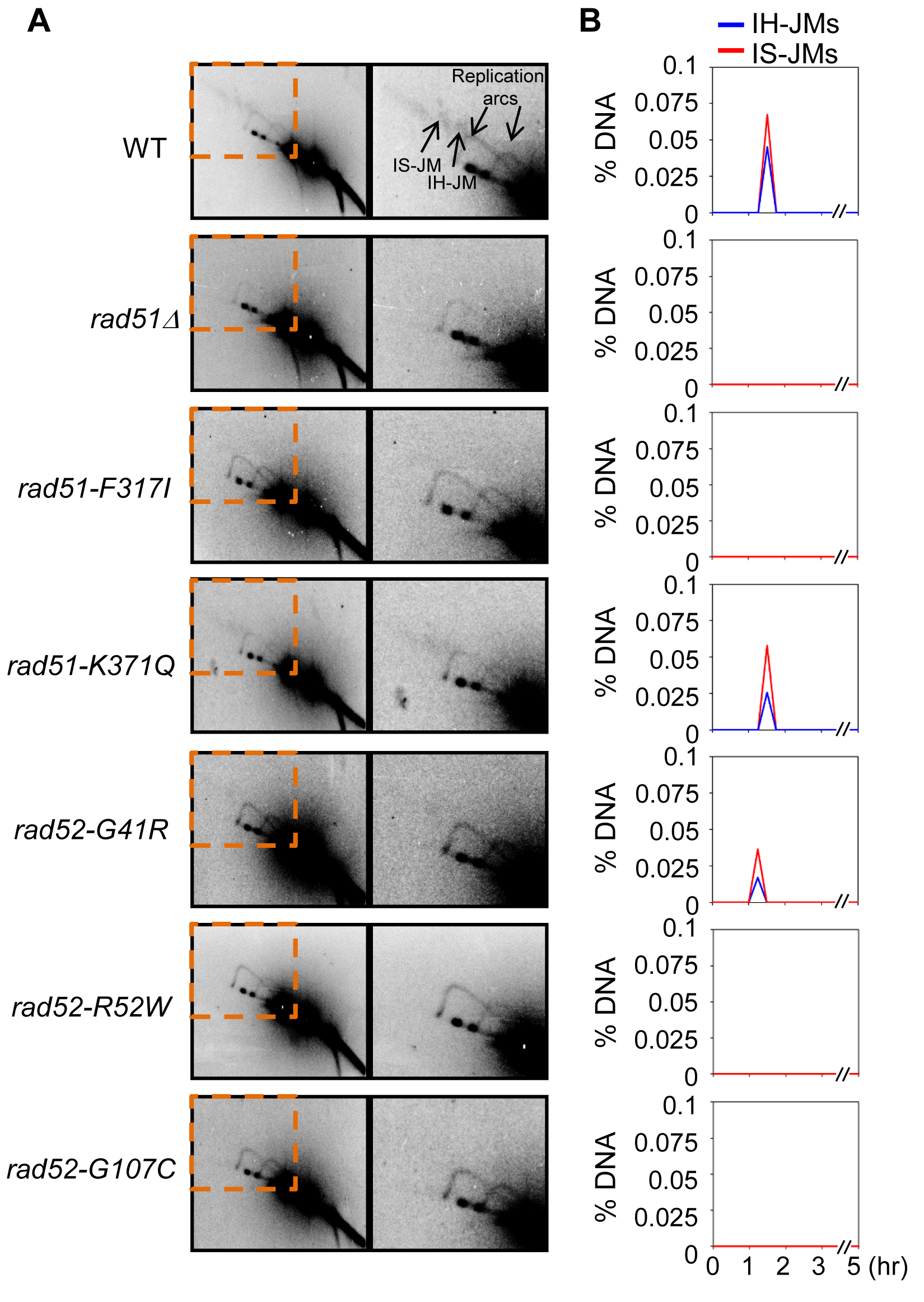

Supplement: S6 Fig — (A) Two-dimensional gel of JM formation over the time course in WT (KKY940), rad51Δ (KKY1088), rad51-F317I (KKY1086), rad51-K371Q (KKY1091), rad52Δ (KKY1142), rad52-G41R (KKY1143), rad52-R52W (KKY1145), rad52-G107C (KKY655). (B) Quantification of JM formation. (TIF) [file pone.0124152.s006.tif]
