# Supplementary material for: Functional Validation of Rare Human Genetic Variants Involved in Homologous Recombination Using Saccharomyces cerevisiae
Source: PLoS One. 2015 May 4;10(5):e0124152. doi: 10.1371/journal.pone.0124152 (PMC4418691; doi:10.1371/journal.pone.0124152)
Supplement: S1 Table — (DOCX) [file pone.0124152.s007.docx]

**S1 Table. Strain list**

| Strain No. | Genotype* |
| --- | --- |
| KKY940 | *MATa/α lys2::URA3-pGal1/10-SCEI/", HIS4::LEU2(I-SceI-WT)/his4::LEU2(I-SceI-mt)* |
| KKY721 | *MATa/α lys2::URA3-pGal1/10-SCEI/", HIS4::LEU2(I-SceI-WT)/his4::LEU2(I-SceI-mt), Rad51-hygB/"* |
| KKY1088 | *MATa/α lys2::URA3-pGal1/10-SCEI/", HIS4::LEU2(I-SceI-WT)/his4::LEU2(I-SceI-mt), rad51Δ::HygB/"* |
| KKY1086 | *MATa/α lys2::URA3-pGal1/10-SCEI/", HIS4::LEU2(I-SceI-WT)/his4::LEU2(I-SceI-mt), rad51-F317I::HygB /"* |
| KKY 1091 | *MATa/α lys2::URA3-pGal1/10-SCEI/", HIS4::LEU2(I-SceI-WT)/his4::LEU2(I-SceI-mt), rad51-K371Q::HygB /"* |
| KKY1978 | *MATa/α lys2::URA3-pGal1/10-SCEI/", HIS4::LEU2(I-SceI-WT)/his4::LEU2(I-SceI-mt), rad52::HygB/"* |
| KKY1142 | *MATa/α lys2::URA3-pGal1/10-SCEI/", HIS4::LEU2(I-SceI-WT)/his4::LEU2(I-SceI-mt), rad52Δ::HygB/"* |
| KKY1143 | *MATa/α lys2::URA3-pGal1/10-SCEI/", HIS4::LEU2(I-SceI-WT)/his4::LEU2(I-SceI-mt), rad52-G41R::HygB /"* |
| KKY1145 | *MATa/α lys2::URA3-pGal1/10-SCEI/", HIS4::LEU2(I-SceI-WT)/his4::LEU2(I-SceI-mt), rad52-R52W::HygB /"* |
| KKY655 | *MATa/α lys2::URA3-pGal1/10-SCEI/", HIS4::LEU2(I-SceI-WT)/his4::LEU2(I-SceI-mt), rad52-G107C::HygB /"* |
| KKY2038 | *MATa/α rad51::HygB* |
| KKY2039 | *MATa/α rad52::HygB* |
| KKY2050 | *MATa/α rad51::hisG / rad51-F317I::HygB* |
| KKY2040 | *MATa/α rad51::HygB / rad51-K371Q::HygB* |
| KKY2041 | *MATa/α rad52::HygB / rad52-G41R::HygB,* |
| KKY2048 | *MATa/α rad52::HygB / rad52-R52W::HygB* |
| KKY2064 | *MATa/α rad52::HygB / rad52-G107C::HygB* |
| KKY1979 | *MATa/α rad51-F317I::HygB / rad51-K371Q::HygB* |
| KKY1980 | *MATa/α rad51-F317I::HygB / rad52-G41R::HygB* |
| KKY1981 | *MATa/α rad51-F317I::HygB / rad51-R52W::HygB* |
| KKY2003 | *MATa/α rad51-F317I::HygB / rad51-G107C::HygB* |
| KKY1982 | *MATa/α rad51-K371Q::HygB / rad52-G41R::HygB* |
| KKY1983 | *MATa/α rad51-K371Q::HygB / rad52-R52W::HygB* |
| KKY1984 | *MATa/α rad51-K371Q::HygB / rad52-G107C::HygB* |
| KKY1985 | *MATa/α rad52G41R::HygB / rad52-R52W::HygB* |
| KKY1986 | *MATa/α rad52G41R::HygB / rad51-G107C::HygB* |
| KKY2004 | *MATa/α rad52-R52W::HygB / rad52-G107C::HygB* |

* All strains are homozygous for *leu2::hisG*, *ho::hisG* and *ura3Δ*(*sma-pst*). WT, wild-type; mt, mutated I-*Sce*I recognition site that is not cleaved by I-*Sce*I *in vivo* as described [40].
