# Supplementary material for: Functional Validation of Rare Human Genetic Variants Involved in Homologous Recombination Using Saccharomyces cerevisiae
Source: PLoS One. 2015 May 4;10(5):e0124152. doi: 10.1371/journal.pone.0124152 (PMC4418691; doi:10.1371/journal.pone.0124152)
Supplement: S2 Table — (DOCX) [file pone.0124152.s008.docx]

**S2 Table. Non-synonymous SNPs in functional domains of human *RAD51* and *RAD52***

| **Gene** | **SNP** | **Position** | **Domain** | **SIFT** | **PolyPhen** | **Amino acid in yeast** |
| --- | --- | --- | --- | --- | --- | --- |
| Rad51 | rs80233386 | Val52Gly | recomb_RAD51 | Deleterious | Possibly damaging | Val |
|  | rs45623838 | Pro56Ser | recomb_RAD51 | Tolerated | Possibly damaging | Pro |
|  | rs7174493 | Leu109Val | recomb_RAD51/Rad51_DMC1_radA | Tolerated | Possibly damaging | Leu |
|  | rs77029343 | Glu169Lys | recomb_RAD51/Rad51_DMC1_radA | Deleterious | Possibly damaging | Glu |
|  | rs76693779 | Met210Leu | recomb_RAD51/Rad51_DMC1_radA | Tolerated | Benign | Met |
|  | rs11544205 | Ala240Ser | recomb_RAD51/Rad51_DMC1_radA | Tolerated | Benign | Ala |
|  | rs2229876ǂ | Phe259Ile | recomb_RAD51/Rad51_DMC1_radA | Deleterious | Possibly damaging | Phe |
|  | rs1056742ǂ | Lys313Gln | recomb_RAD51/Rad51_DMC1_radA | Deleterious | Possibly damaging | Lys |
| Rad52 | rs35008685ǂ | Gly59Arg | Rad52_Rad22 | Deleterious | Probably damaging | Gly |
|  | rs11571421ǂ | Arg70Trp | Rad52_Rad22 | Deleterious | Probably damaging | Arg |
|  | rs1131839 | Asn100Lys | Rad52_Rad22 | Deleterious | Possibly damaging | Asn |
|  | rs77010242ǂ | Gly125Cys | Rad52_Rad22 | Deleterious | Probably damaging | Gly |

ǂ Selected SNPs for experiment
